# Deep learning-based optical coherence tomography and retinal images for detection of diabetic retinopathy: a systematic and meta analysis

**DOI:** 10.3389/fendo.2025.1485311

**Published:** 2025-03-18

**Authors:** Zheng Bi, Jinju Li, Qiongyi Liu, Zhaohui Fang

**Affiliations:** ^1^ Department of Endocrinology, The First Affiliated Hospital of Anhui University of Traditional Chinese Medicine, Hefei, Anhui, China; ^2^ First Clinical Medical College, Anhui University of Traditional Chinese Medicine, Hefei, Anhui, China; ^3^ Xin ‘an Medical and Chinese Medicine Modernization Research Institute, Hefei Comprehensive National Science Center, Hefei, Anhui, China

**Keywords:** meta analysis, deep learning, diabetic retinopathy, image detection, optical coherence tomography

## Abstract

**Objective:**

To systematically review and meta-analyze the effectiveness of deep learning algorithms applied to optical coherence tomography (OCT) and retinal images for the detection of diabetic retinopathy (DR).

**Methods:**

We conducted a comprehensive literature search in multiple databases including PubMed, Cochrane library, Web of Science, Embase and IEEE Xplore up to July 2024. Studies that utilized deep learning techniques for the detection of DR using OCT and retinal images were included. Data extraction and quality assessment were performed independently by two reviewers. Meta-analysis was conducted to determine pooled sensitivity, specificity, and diagnostic odds ratios.

**Results:**

A total of 47 studies were included in the systematic review, 10 were meta-analyzed, encompassing a total of 188268 retinal images and OCT scans. The meta-analysis revealed a pooled sensitivity of 1.88 (95% CI: 1.45-2.44) and a pooled specificity of 1.33 (95% CI: 0.97-1.84) for the detection of DR using deep learning models. All of the outcome of deep learning-based optical coherence tomography ORs ≥0.785, indicating that all included studies with artificial intelligence assistance produced good boosting results.

**Conclusion:**

Deep learning-based approaches show high accuracy in detecting diabetic retinopathy from OCT and retinal images, supporting their potential as reliable tools in clinical settings. Future research should focus on standardizing datasets, improving model interpretability, and validating performance across diverse populations.

**Systematic Review Registration:**

https://www.crd.york.ac.uk/PROSPERO/, identifier CRD42024575847.

## Introduction

Diabetic retinopathy (DR) is one of the most common microvascular complications of diabetes and a leading cause of blindness in adults worldwide (1). As the prevalence of diabetes continues to rise globally, the incidence of DR is also increasing significantly. Retinal vascular abnormalities, which are hallmarks of DR, gradually lead to a decline in patients’ vision and, in severe cases, can cause blindness (1, 2). Given the current medical capabilities, the disease cannot be completely cured; treatment focuses on maintaining the patient’s existing level of vision. If DR can be diagnosed and treated early, in most cases, patients’ vision can be preserved. Early detection and timely treatment of DR are crucial for preventing vision loss (3).

Traditionally, the detection of DR relies on ophthalmologists’ manual evaluation of retinal images (4). However, this method is time-consuming, labor-intensive, and subject to variability due to the experience and subjective judgment of the evaluators, leading to inconsistent detection outcomes (5). Currently, most ophthalmologists still use traditional methods to diagnose diabetic retinopathy (DR) by analyzing the presence and types of abnormalities in retinal images. Microaneurysms (MIA), hemorrhages (HEM), soft exudates (SOX), and hard exudates (HEX) are the four most common types of lesions (3–5).

Manual detection of diabetic retinopathy (DR) images presents several issues. First, interpreting DR images requires trained ophthalmologists, but in underdeveloped countries, there is a severe shortage of ophthalmologists, leading to many patients being unable to receive timely screening and treatment (6). Additionally, the cost of DR examinations is high, making it unaffordable for many patients and causing them to miss the opportunity for early intervention. These issues contribute to the high prevalence and risk of blindness associated with DR (7). Since timely detection is crucial in preventing vision loss, scientists and engineers have been working to design automated methods to achieve accurate and rapid diagnosis and treatment. Automated methods not only address the shortage of human resources but also significantly reduce the cost of screening, benefiting more patients (8). In recent years, with the rapid development of machine learning (ML) and artificial intelligence (AI) technologies, ML models trained on a large number of fundus images have achieved high accuracy in automated DR classification (9). These models can quickly and efficiently analyze large volumes of images, allowing for a substantial number of screenings to be completed in a short time. To further improve detection performance, substantial effort has been invested in developing automated methods that are both efficient and cost-effective. These methods not only consider the accuracy of detection but also emphasize ease of use and cost control, making them more suitable for implementation in resource-limited settings (10).

Recently, advancements in optical coherence tomography (OCT) and retinal imaging technology have provided high-resolution image data for the early detection of DR (9, 10). OCT technology can generate detailed three-dimensional images of the retina, revealing subtle lesion features, allowing for detection of abnormalities at an early stage of the disease. These high-resolution image data greatly enhance the performance of automated detection systems, enabling more accurate identification and classification of DR lesions, thereby providing timely and effective treatment recommendations for patients (11). These images can capture minute changes in the retina, enabling more accurate detection of DR. With the rapid development of deep learning technology, significant breakthroughs have been achieved in the field of computer vision. Deep learning algorithms, particularly convolutional neural networks (CNNs), have demonstrated exceptional performance in image recognition and classification tasks and have been widely applied in medical image analysis. Kazakh-British et al. (11)conducted experimental research using relevant processing pipelines to extract arteries from fundus images and then trained CNN models to identify lesions (9). Alexandr et al. (12) compared two widely used classical designs (DenseNet and ResNet) with a new enhanced structure (EfficientNet) in their other work. Previous studies have shown that deep learning-based models can automatically analyze OCT and retinal images, accurately identifying and classifying different stages of DR (13).

Despite numerous studies exploring the application of deep learning in DR detection, their results and conclusions often vary, and a unified perspective has yet to emerge. Therefore, there is a need for a systematic review and meta-analysis to comprehensively evaluate the effectiveness of deep learning-based OCT and retinal image analysis for DR detection, clarifying its clinical value and future directions.

This study aims to systematically review and meta-analyze existing research to assess the accuracy and reliability of deep learning models in detecting DR from OCT and retinal images and to identify key factors influencing detection performance. Through this research, we hope to provide scientific evidence for clinical practice and promote the application and popularization of deep learning technology in ophthalmic diagnostics.

## Materials and methods

Reporting of this review and meta-analysis followed the PRISMA checklist. The study protocol was registered after the initial screening stage. The design of the inclusion and exclusion criteria of this study was based on the five main principles of the Participant-Intervention-Comparator-Outcomes-Study (PICOS) design search principle (14). Our PICO question was as follows: In deep learning applications developed based on retinal images for early screening of diabetic retinopathy (Participants), how does DL (Intervention) compare with traditional landmarks by a single expert or with scripted eye care provider referral and education (Control) in terms of accuracy (Outcome). The systematic evaluation program is registered on the International Prospective Systems Evaluation website (PROSPERO-CRD42024575847).

### Inclusion criteria

The included patients all had diabetic retinopathy, regardless of age, sex, or race. The control group received conventional basic treatment (e.g., scripted eye care provider referral and education). The treatment group was treated with deep learning-based optical coherence tomography and retinal images (color fundus photography). The primary outcome indicators were as follows: diabetic eye exam completion rate, the proportion of participants who completed follow-through with an eye care provider, and DR classification accuracy. The types of included literature were randomized controlled trials (RCTs) and observational studies, with no restrictions on language, blinding, or allocation concealment requirements. Any study approved by the local institution was included in the scope of this study and registered in the international database.

### Exclusion criteria

Self-control studies, case reports, literature reviews, duplicate publications, experience summaries, animal experiment research, studies with incomplete data, studies involving patients with other diseases, studies lacking clear diagnostic or efficacy evaluation standards, and studies combining other therapies different from the control group were excluded.

### Information sources

We systematically screened five electronic databases(Cochrane library, PubMed, Embase, IEEE Xplore, Web of science) for studies published up January 2017 to July 2024. Search terms included Coherence Tomography, Optical, Optical Coherence Tomography, OCT Tomography, Tomography, OCT (Spectral Domain OCT (SD-OCT). This allowed for a high-resolution 3D imaging of the retinal layers and provided detailed information for the deep learning model analysis), Diabetic Retinopathies, Retinopathies, Diabetic, Retinopathy, Diabetic, Deep learning-based,Deep learning. A two-pronged search strategy, combining the technique of interest (AI, CNN, DL, etc.) and the diagnostic target, was applied. The best effort was made to ensure the comprehensiveness of the preliminary search work so as not to lose valuable research data. According to the search modes of different databases, keywords could be combined with free words for a comprehensive search.

### Data collection, items, and study selection

Based on the electronic database search strategy outlined above, two researchers conducted searches in both Chinese and English electronic databases. They used EndNote X7 software to identify and remove duplicate studies, integrated the search results from the different databases, created an information database, and downloaded the full texts of the relevant studies. Subsequently, two researchers independently performed preliminary screening and extracted data according to a pre-defined table. They cross-checked and reviewed the extracted data, recorded the reasons for excluding each study, and consulted third-party experts to resolve differing opinions and reach a final decision. The data extraction encompassed fundamental details from the included studies (e.g., first author and publication year), pertinent information about the experimental and control groups (such as case numbers, intervention measures, and outcome indicators), and the study design along with quality assessment data (including randomization methods, blinding procedures, allocation concealment, completeness of outcome data, selective reporting, and other sources of bias). The search strategy was as follows: (((Coherence Tomography, Optical[MeSH Terms]) OR Optical Coherence Tomography[MeSH Terms]) OR OCT Tomography[MeSH Terms]) OR Tomography, OCT[MeSH Terms] AND ((Diabetic Retinopathies[MeSH Terms]) OR Retinopathies, Diabetic[MeSH Terms]) OR (Retinopathy, Diabetic[MeSH Terms]) AND (Deep learning-based [MeSH Terms]) OR (Deep learning[MeSH Terms]).

### Quality assessment

The methodological quality of the included studies was assessed using Cochrane’s revised risk of bias tool for randomized trials (RoB 2.0) (15). This evaluation covered various aspects including the randomization process, deviations from intended interventions, missing outcome data, outcome measurement, and the selection of reported result areas. Each evaluation module consists of several signal questions, with possible responses being: Y (yes), PY (probably yes), PN (probably no), N (no), and NI (no information). Risk of bias was assessed independently by two reviewers, who discussed their findings in case of disagreement to come to aconsensus. We do not provide further guidance as to the certainty of the evidence (e.g., using any kind of grading), but provide descriptive statistics of the individual and overall risk of bias together with meta-analytic estimates.

### Statistical methods and data synthesis

First, the authors used RevMan5.4 software to analyze the publication bias of the literature. Second, for the direct comparison results, the authors used Stata17.0 software for data merging, statistical analysis and meta-analysis. In Stata17.0, the meta package was used to perform meta-analysis. The relevant commands were executed to analyze data with both fixed-effect and random-effects models. The meta package provided functionalities for computing heterogeneity statistics, generating forest plots, and creating funnel plots. For meta-regression analysis to explore sources of heterogeneity, the metareg package was utilized with specific covariates. The analysis involved using these packages to compare different interventions and to map network meta-analysis results with random-effects model data. Significance was determined using P < 0.05 and 95% confidence intervals (95% CIs). For efficacy analysis, odds ratios (OR) were used for count data, while measurement data employed either the weighted mean difference or the standardized mean difference (mean difference, MD). Each effect size was reported with a 95% CI (16).

### Assessment of heterogeneity

The heterogeneity was graded using I^2^ according to the recommendations of the Cochrane Handbook (17). Cochrane’s Q test was used to detect whether there was a significant difference in effect sizes between studies. The Q statistic followed a chi-squared distribution, and the P-value was used to determine the significance of heterogeneity. If the P-value was significant (typically < 0.05), it indicated substantial heterogeneity. The I²index statistic represented the percentage of total variation due to heterogeneity. The I²index ranged from 0% to 100%, with higher values indicating greater heterogeneity. Generally, 0% to 25% suggested low heterogeneity, 25% to 50% indicated moderate heterogeneity, 50% to 75% indicated substantial heterogeneity, and 75% to 100% indicated considerable heterogeneity.

The clinical and methodological heterogeneity of the included studies was evaluated, and the levels of fit of the fixed-effects model and the random-effects model were compared (18). In the absence of significant clinical heterogeneity (P ≥ 0.1, I^2^ ≤ 50%), a fixed-effects model was used for meta-analysis. If there was significant clinical heterogeneity between the results of each study (P < 0.1, I^2^ > 50%), the source of the heterogeneity was first analyzed, the influence of clinical or methodological heterogeneity was excluded and the random-effects model was used for the meta-analysis. When the data provided by the clinical trial could not be meta-analyzed, they were subjected to a descriptive analysis.

### Publication bias

According to the recommendations of the Cochrane Handbook, the RevMan 5.4 software was used to analyze potential publication bias (19). Typically, in the absence of publication bias, a funnel plot should appear symmetrical. If the funnel plot is asymmetrical, it may indicate the presence of publication bias. Egger’s regression test was performed using Stata 17.0 to calculate publication bias. This test assesses the symmetry of the funnel plot through regression analysis to quantitatively detect publication bias. If the intercept of the regression significantly deviates from zero, it suggests the presence of publication bias (20).

## Results

### Study selection and characteristics

**Figure 1:**
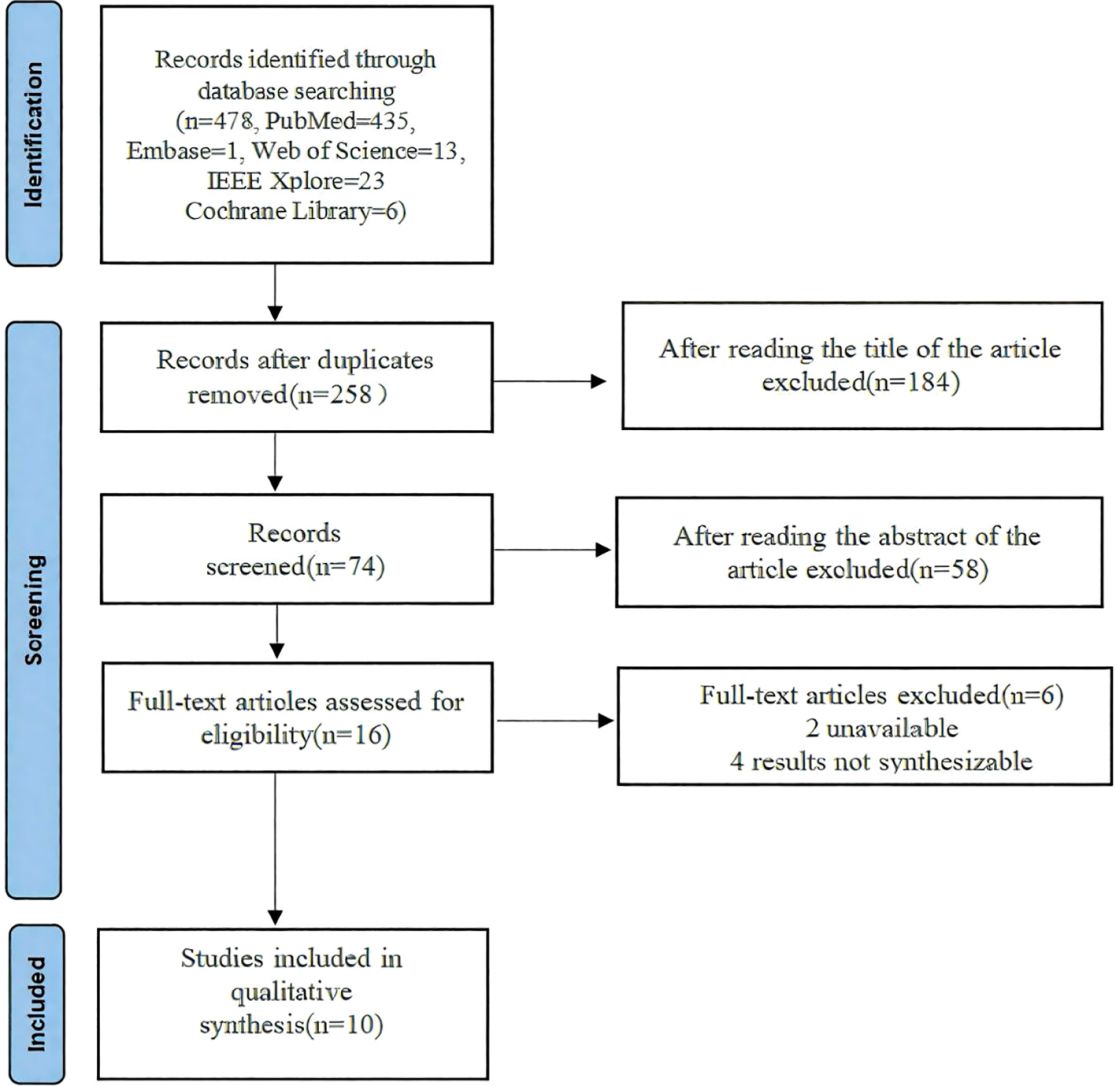
The flow chart of literature screening.

From 478 identified studies, 258 were screened in full texts, and 10 studies were eventually included in our review and meta analysis. The report flowchart is shown in **Figure 1**. The treatment group included 8 Artificial intelligence (AI)-based algorithm, Inoveon Diabetic Retinopathy (DR-3DT) system and Nonmydriatic ultra-widefield (NM UWF) screening. The basic characteristics of the included studies are shown in **Tables 1** and **2**. Three studies focused on the analysis of retinal images, which used own data. Seven studies used publicly available data from cohort studies. 86% of the DL models were built using CNN algorithms, with one study using Inception-V4 and five studies using Inception-V3. The outcome measures of the studies all evaluated the accuracy and sensitivity of DL in monitoring diabetic retinopathy, furthermore, ETDRS macular edema stage the “gold standard” for the evaluation of diabetic retinopathy.

**Table 1:** Characteristics of the selected studies included in the systematic review and meta-analysis.

Author(year)	Type of study	Treatment measures	Sample size (invention/control)	Age (years)	Outcome indicators	Treatment time	Reference	Jadad Scale
Risa M 2024	Randomized control trial	Artificial intelligence (AI)	164(81/83)	8-21	diabetic eye exam completion rate; the proportion of participants	6 months	(21)	4
Selina L 2023	Randomized control trial	Nonmydriatic ultra-widefield (NM UWF) screening	658(335/323)	30-61	the proportions of AED between groups	12 months	(22)	3
Stephen R 2002	Observational study	Inoveon Diabetic Retinopathy (DR-3DT) system	290(145/145)	–	Accuracy (sensitivity, specificity, predictive values) of the digital system	–	(23)	4
Huma N 2022	Randomized control trial	Automated unsupervised deep learning	Data set: 3662	24-74	Accuracy (sensitivity, specificity, predictive values)	–	(24)	3
Wang Y 2021	Observational study	Artificial intelligence (AI)-based algorithm	Data set:12252	–	Accuracy (sensitivity, specificity, predictive values)	–	(25)	3
Alwakid G 2023	Observational study	Artificial intelligence (AI)-based algorithm	Data set:9952	–	Accuracy (sensitivity, specificity, predictive values)	–	(26)	3
Mehboob A 2022	Observational study	Artificial intelligence (AI)-based algorithm	Data set:96213	–	Accuracy (sensitivity, specificity, predictive values)	–	(27)	4
Li F 2022	Observational study	Artificial intelligence (AI)-based algorithm	Data set:8739	–	Accuracy (sensitivity, specificity, predictive values)	–	(28)	3
Surya J 2023	Randomized control trial	Artificial intelligence (AI)-based algorithm	723(382/341)	35-65	Accuracy (sensitivity, specificity, predictive values)	6 months	(29)	3
Mansour R 2017	Observational study	Artificial intelligence (AI)-based algorithm	Data set:35126	–	Accuracy (sensitivity, specificity, predictive values)	–	(30)	4
Nunez d 2022	Observational study	Artificial intelligence (AI)-based algorithm	Data set:20489	53-67	Accuracy (sensitivity, specificity, predictive values)	–	(3)	2

**Table 2:** Characteristics of the selected studies (Artificial intelligence (AI)-based algorithm).

Author (year)	Type of machine learning models	Imaging modality	Imaging pattern	Accuracy of result	Sensitivity of result	Reference
Risa M 2024	autonomous AI system	fundus images	–	100%	78.9%	(21)
Selina L 2023	–	OCT images	100°, 200°	99.1%	–	(22)
Stephen R 2002	–	fundus images	30°, 1152 × 1152 pixels	98.2%	89.7%	(23)
Huma N 2022	the fuzzy clustering method, deep embedded clustering, and k-means for generalizability			98.66%	–	(24)
Wang Y 2021	CNN	color fundus photography	–	90.6%	90.6%	(25)
Alwakid G 2023	Inception-V3, CNN	high-resolution retinal pictures	3216 × 2136 pixels	98%	–	(26)
Mehboob A 2022	RFT, CNN	color fundus photography	90°, 180°	83.78%	78.55%	(27)
Li F 2022	CNN	color fundus photography	–	90.21%	93.24%	(28)
Surya J 2023	CNN	underwent fundus photographs	45°	89.75%	83.33%	(29)
Mansour R 2017	DNN, CNN	color fundus photography	–	90.15%	91.3%	(30)
Nunez d 2022	CNN	color retinal images	40°	92.56%	91.22%	(3)

CNN, Convolutional Neural Network; Inception-V3, CNN, Inception-V3 Convolutional Neural Network; RFT, Random Forest Tree; Inception-V4, Inception-V4 Convolutional Neural Network; Dr Noon AI, Doctor Noon Artificial Intelligence; DNN, Deep Neural Network; CNN, Convolutional Neural Network; VISUHEALTH-AI DR, VISUHEALTH Artificial Intelligence for Diabetic Retinopathy.

Notably, many studies employed multiple test datasets. The reference test in the training dataset was established by two experts in 7 studies.

### Risk of bias and applicability concerns

**Figure 2:**
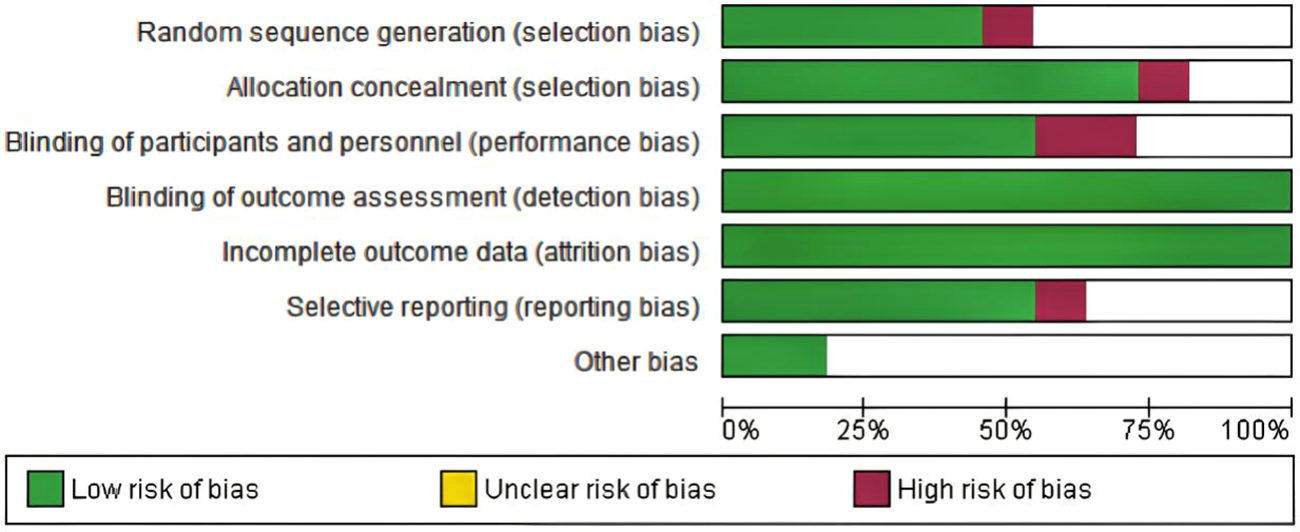
Risk of bias graph in the included studies.

**Figure 3:**
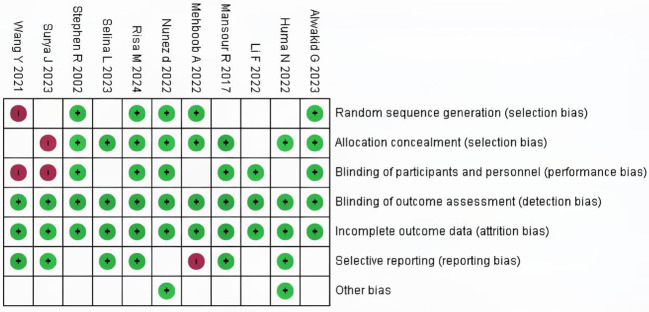
Risk of bias summary in the included studies.

Among the 10 included studies, 4 were double-arm randomized controlled trials (RCTs) and 6 were observational studies. In the 7 evaluation modules, 5 were rated as low risk. In the assessment of missing outcome data and data integrity, all 10 studies were rated as low risk according to the RoB 2.0 evaluation results, indicating good quality and complete data in the included literature. However, two studies were assessed as high risk regarding participant details because they used cohort reporting and did not provide specific information on participant age and other demographics. The risk of research bias is expressed as a percentage of all the included studies, as shown in **Figures 2** and **3**.

### Meta-analysis

**Figure 4:**
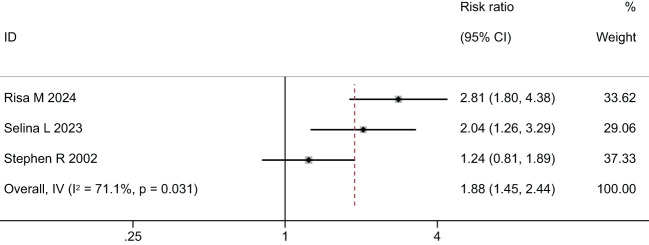
Forest plot of studies reporting the effectiveness of imaging to screen for Diabetic Eye Disease (primary outcome).

**Figure 5:**
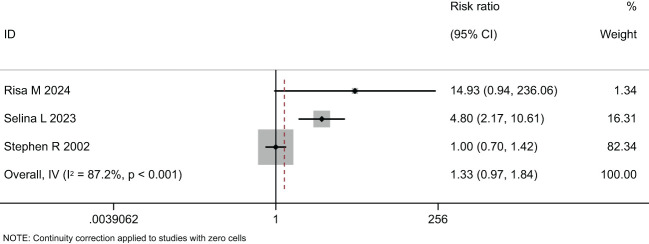
Forest plot of studies reporting the effectiveness of imaging to screen for Diabetic Eye Disease (secondary outcome).

Two meta-analyses were performed, one synthesizing the effectiveness of imaging to screen for Diabetic Eye Disease (**Figure 4**) and one on the proportion of Deep-learning-based automatic computer-aided diagnosis system for diabetic retinopathy (**Figure 5**).

In the comparison of the accuracy of diagnosing diabetic retinopathy using deep learning-based optical coherence tomography and retinal images, the results were shown in **Figures 4**, **5**, and **Table 3**. A random-effects model was used when I2 > 50. The forest plot results showed that, compared to standard care, autonomous artificial intelligence improved the completion rate of diabetic eye exams in adolescents with diabetes [OR = 1.88, 95% CI = (1.45, 2.44), p = 0.031]. The overall detection accuracy with the assistance of artificial intelligence also showed significant improvement compared to traditional methods [OR = 1.33, 95% CI = (0.97, 1.84), p < 0.001]. All of the outcome of deep learning-based optical coherence tomography had ORs ≥0.785, indicating that all included studies with artificial intelligence assistance produced good boosting results (**Table 3**).

**Table 3:** The outcome of deep learning-based optical coherence tomography (OR,95%CI).

Id	OR lower	OR-OR lower	OR	OR upper-OR	OR upper	P-value
Huma N 2022 (24)	0.9753	0.0113	0.9866	0.0061	0.9927	<0.001
Wang Y 2021 (25)	0.9016	0.0207	0.9223	0.0347	0.957	<0.001
Alwakid G 2023 (26)	0.808	0.073	0.881	0.106	0.987	<0.001
Mehboob A 2022 (27)	0.7308	0.0542	0.785	0.0528	0.8378	<0.001
Li F 2022 (28)	0.916	0.009	0.925	0.011	0.936	<0.001
Surya J 2023 (29)	0.7755	0.0745	0.85	0.0745	0.9245	<0.001
Mansour R 2017 (30)	0.9015	0.04	0.9415	0.0378	0.9793	<0.001

Heterogeneity was assessed using funnel plots and the Egger test. The funnel plots in **Supplementary Figures S1** and **S3** were relatively symmetrical, with the effect sizes of the studies evenly distributed around the overall effect size. Egger’s test, a regression test used for quantitatively assessing publication bias, showed p-values of 0.686 (> 0.05) in **Supplementary Figure S2** and 0.569 (> 0.05) in **Supplementary Figure S4**, indicating that the symmetry of the funnel plots was not significant and the likelihood of publication bias was low, suggesting no heterogeneity.

## Discussion

Currently, the assessment of the severity of diabetic retinopathy in patients heavily relies on manual interpretation of retinal fundus images, which poses significant challenges (31). Therefore, automated image grading systems play a crucial role in the early diagnosis and evaluation of these vision-threatening diseases. For example, deep learning algorithms and image processing techniques can analyze large volumes of fundus images, providing consistent and highly accurate diagnostic results, reducing human error, and improving diagnostic accuracy (32–34). By regularly collecting and analyzing patients’ fundus images, automated image grading systems can continuously monitor the progression of diabetic retinopathy, assisting doctors in timely adjusting treatment plans to achieve the best therapeutic outcomes (34). Multiple studies (35–37) have shown that deep learning algorithms can be used to generate expert-level grading diagnoses for retinal fundus images. However, these methods often achieve good performance at the expense of increased time complexity. Due to the same input image size in these independent models, the robustness of their classification is relatively poor. Therefore, this study employs a systematic review and meta-analysis to analyze the role of deep learning-based optical coherence tomography and retinal images in the detection of diabetic retinopathy.

The results of this meta-analysis confirmed that, compared to standard care, autonomous artificial intelligence improved the completion rate of diabetic eye exams in adolescents with diabetes [OR=1.88, 95% CI=(1.45, 2.44), p=0.031]. Risa M et al. (21) were the first to assess the role of artificial intelligence in narrowing the care gap among racially and ethnically diverse adolescent diabetic patients. The study indicated that closing the care gap for diabetic eye exams, as measured by MIPS and HEDIS quality indicators, was a crucial component of value-based care. The results suggested that autonomous artificial intelligence could help meet these historically challenging benchmarks, particularly among racially/ethnically diverse and resource-limited youth. Li et al. (28) confirmed that, although deep learning (DL) detection often showed larger deviations at points such as the porion, subspinale, gonion, articulare, and anterior nasal spine, DL might not exceed expert detection accuracy but could clearly assist both regular and experienced examiners in landmark detection. Training models on larger datasets might have eventually helped achieve or surpass expert accuracy. The results indicated that DL models included in the studies achieved an accuracy above 83% for identifying diabetic retinopathy. A total of 71% of established DL research models had detection accuracies exceeding 90%. Mehboob A et al. (27) proposed a DL architecture consisting of three phases: image pre-processing, feature extraction, and classification. Deep convolutional networks (CNNs) were trained to extract deep features. Heat maps extracted from the proposed framework highlighted the presence of any exudates, microaneurysms, hemorrhages, cotton wool spots, or new vessels, indicating feature extraction from the affected region and achieving high accuracy. Deep CNNs could take unknown images as input and extract problem-specific features, thereby generating an appropriate response. The results showed that the proposed technique outperformed existing ones in terms of sensitivity. Even with a lighter CNN architecture, it demonstrated competitive accuracy. Moreover, among ensemble-based architectures, the proposed framework achieved the highest accuracy using average pooling when trained on an augmented dataset. F. Mansour Romany (30)used deep convolutional networks to classify data into normal and diseased categories with an accuracy of 97.93%.

Common deep learning ensemble algorithm classifiers include Random Forest, Support Vector Machines (SVM), Neural Networks, K-Nearest Neighbors (KNN), Multilayer Perceptrons, Naive Bayes, Decision Trees, and Logistic Regression. In 2021, an ensemble-based machine learning algorithm was proposed (38), which combined three different classifiers: Random Forest, Support Vector Machines (SVM), and Neural Networks, with a meta-classifier for decision-making. This ensemble-based approach enhanced the robustness and performance of the algorithm. The algorithm was tested on the Messidor dataset and achieved an accuracy of 0.75. Another ensemble-based algorithm for diabetic retinopathy screening was proposed by Nagi, A in 2021 (39). This algorithm employed a two-stage classifier, where the first stage consisted of outputs from six classifiers: SVM, KNN, Multilayer Perceptron, Naive Bayes, Decision Trees, and Logistic Regression, followed by a second stage using a Neural Network to make the final decision based on the classifier outputs. The algorithm achieved a test accuracy of 76.40% on the Messidor dataset. In 2020, an ensemble-based deep neural network architecture was established. This model used ResNet (40) and leveraged four ResNets to perform binary classification among five categories of diabetic retinopathy: normal vs. mild DR, normal vs. moderate DR, normal vs. severe DR, and normal vs. proliferative DR. The results from each classifier in Stage 1 were then processed by an AdaBoost classifier in Stage 2 to obtain the final classification results. The algorithm was evaluated on the Kaggle dataset APTOS 3662 retinal images, resulting in an accuracy of 61.9%.

The study results indicated that to enhance the accuracy of artificial intelligence in diabetic retinopathy detection and assessment, an automated algorithm should have followed a two-step strategy (41). The first method involved automatically defining the acceptability of retinal images to determine if they qualified for automatic grading, and then only applying the automated algorithm if the retinal images passed the acceptability test. The second method suggested that to ensure global applicability of automatic grading, the development of automated algorithms should have used images that reflected the specific acquisition conditions in real-world programs, allowing the model to understand and leverage these unique characteristics. Among the most commonly used DL models in research, CNN included two different convolutional neural network (CNN) architectures: Inception-v3 and Inception-v4 (42). These architectures had significant differences in design and performance. Inception-v3 enhanced feature extraction capabilities mainly through improved Inception modules, which included multiple parallel convolutional and pooling layers, as well as 1x1 convolutions to reduce computational complexity. It also introduced batch normalization and separable convolutions to accelerate training and improve efficiency. In contrast, Inception-v4 built upon Inception-v3 by integrating residual networks (Residual Networks), introducing Inception-ResNet and Reduction-ResNet modules (43–45). These improvements gave Inception-v4 deeper network layers and better feature extraction capabilities, while residual connections addressed gradient vanishing issues in deep networks, enhancing training stability. Although Inception-v3 performed excellently in various computer vision tasks, Inception-v4 generally offered higher accuracy and faster training speed. Li et al. (28) developed an improved Inception-v4 network based on stem, inception, and reduction modules, and created an ensemble of five classification model instances based on this Inception-v4 network. Its performance level was comparable to or exceeded that of ophthalmologists, achieving excellent results on the primary dataset used. The detection accuracy was comparable to Inception-v3, but its responsiveness was notably higher than other Inception-v3-based DL models (25–27).

However, as all studies tested in this same dataset (and most also trained on this dataset), we likely have high comparability but limited generalizability. Future studies should aim to test DL models on broad data, demonstrating robustness and generalizability. This review and the included studies have a number of limitations. First, the precision and recallresults for some types of lesions in our study, which we mentioned above, were limited. More training data for these lesions should becollected to improve the performance of our model. Second, The established dataset was not necessarily a good representation of data from screening programs in clinical practice. Thereby, the built dataset was not sufficient to reflect the algorithm’s performance in broader clinical use. Future studies should consider including a wider outcome set and aim to test DL applications comprehensively in other study designs and settings (e.g., observational studies in clinical care, randomized controlled trials).

## Conclusion

DL shows relatively high accuracy for detection of diabetic retinopathy, whether using a self-trained DL model or choosing an established AI model. The majority of studies focused on CNN(Inception-V3)to develop DL models. The results showed that the accuracy of DL models in evaluating diabetic retinopathy was highly consistent across different studies and superior to the control group, with no heterogeneity observed. Further validation with larger datasets is needed, and it is hoped that more randomized controlled trials will be used for model validation, and the true value of using DL in clinical care needs to be demonstrated. Future research should focus on standardizing datasets, improving model interpretability, and validating performance across diverse populations.

## Data Availability

The original contributions presented in the study are included in the article/**Supplementary Material**. Further inquiries can be directed to the corresponding author.

## Supplementary material

Supplementary Figure 1 Funnel plot of studies reporting the effectiveness of imaging to screen for Diabetic Eye Disease (primary outcome).

Supplementary Figure 2 Egger-test of studies reporting the effectiveness of imaging to screen for Diabetic Eye Disease (primary outcome).

Supplementary Figure 3 Funnel plot of studies reporting the effectiveness of imaging to screen for Diabetic Eye Disease (secondary outcome).

Supplementary Figure 4 Egger-test of studies reporting the effectiveness of imaging to screen for Diabetic Eye Disease (secondary outcome).
