# Supplementary figures and images for: Deep learning-based optical coherence tomography and retinal images for detection of diabetic retinopathy: a systematic and meta analysis
Source: Front Endocrinol (Lausanne). 2025 Mar 18;16:1485311. doi: 10.3389/fendo.2025.1485311 (PMC11958191; doi:10.3389/fendo.2025.1485311)

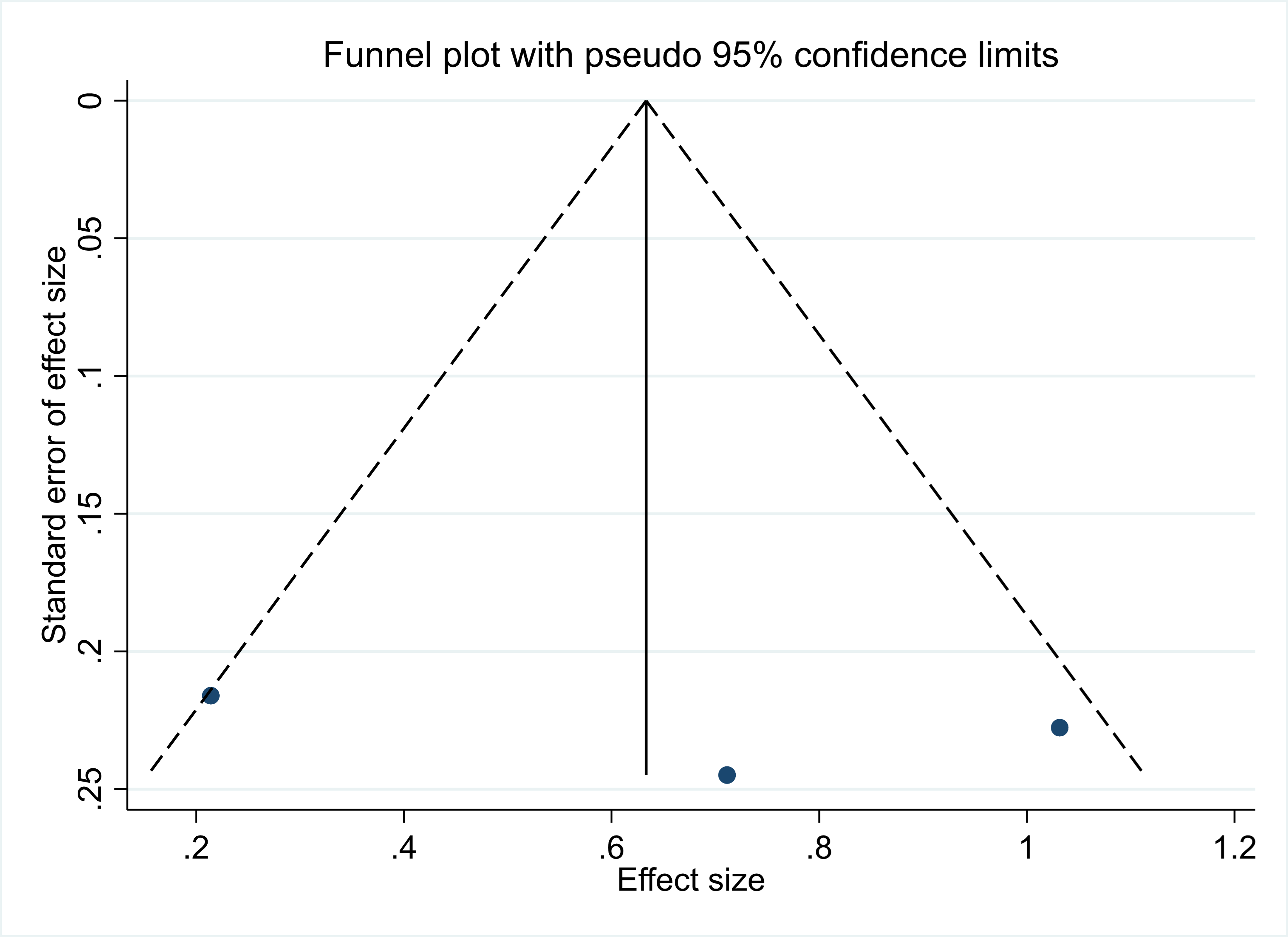

Supplement: Supplementary Figure 1 — Funnel plot of studies reporting the effectiveness of imaging to screen for Diabetic Eye Disease (primary outcome). [file Image1.tif]

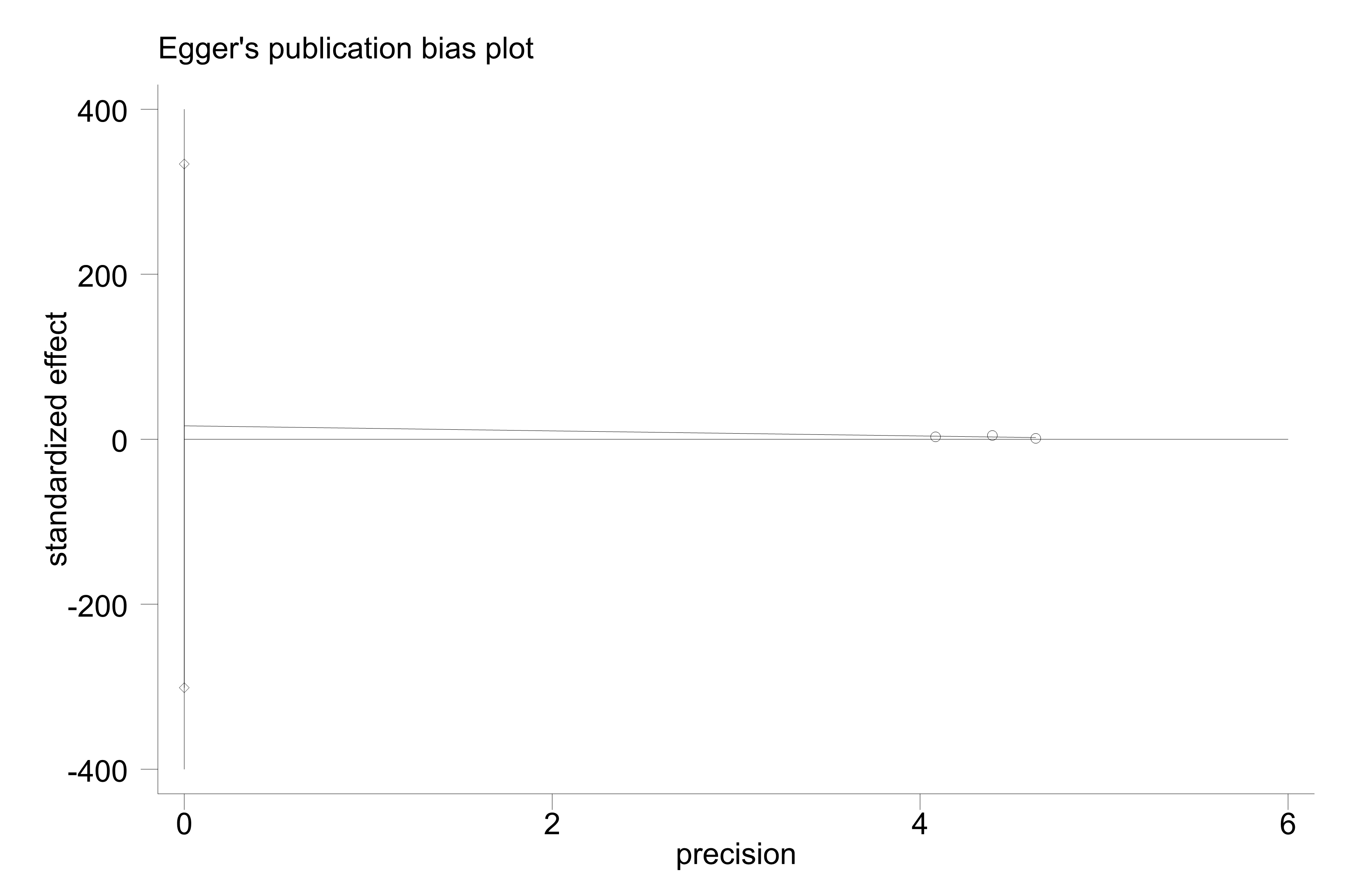

Supplement: Supplementary Figure 2 — Egger-test of studies reporting the effectiveness of imaging to screen for Diabetic Eye Disease (primary outcome). [file Image2.tif]

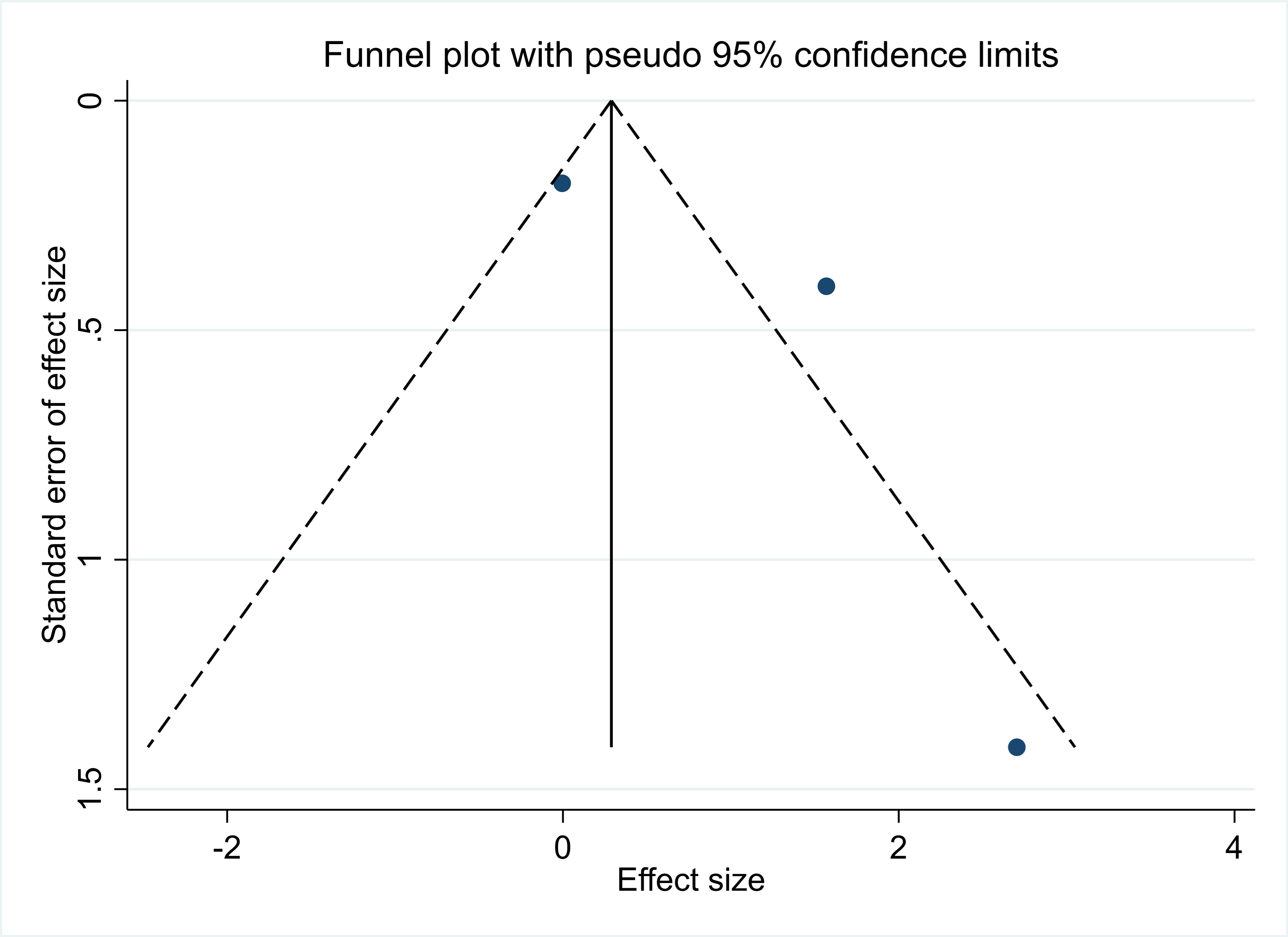

Supplement: Supplementary Figure 3 — Funnel plot of studies reporting the effectiveness of imaging to screen for Diabetic Eye Disease (secondary outcome). [file Image3.tif]

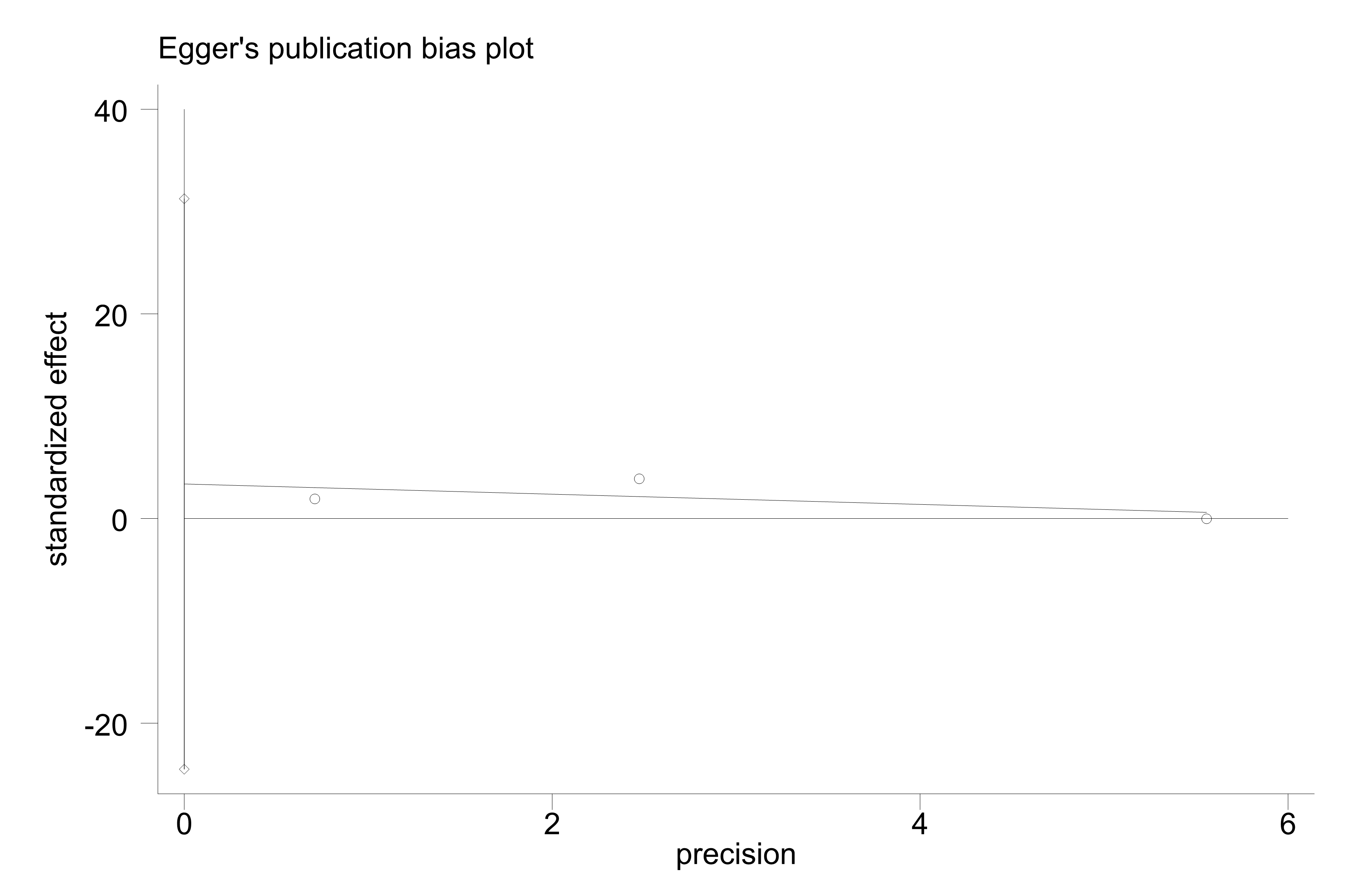

Supplement: Supplementary Figure 4 — Egger-test of studies reporting the effectiveness of imaging to screen for Diabetic Eye Disease (secondary outcome). [file Image4.tif]
